# Seawater Effects on Thermally Aged Ambient Cured Carbon/Epoxy Composites: Moisture Kinetics and Uptake Characteristics

**DOI:** 10.3390/polym15092138

**Published:** 2023-04-29

**Authors:** Vistasp M. Karbhari, Rabina Acharya, SoonKook Hong

**Affiliations:** 1 Department of Civil Engineering, University of Texas Arlington, Arlington, TX 76006, USA; 2 Department of Mechanical and Aerospace Engineering, University of Texas Arlington, Arlington, TX 76006, USA; 3 Department of Mechanical and Naval Architectural Engineering, Naval Academy, Changwon City 440-746, Republic of Korea

**Keywords:** thermal aging, seawater, carbon/epoxy composite, diffusion, deterioration, hygrothermal, relaxation

## Abstract

Carbon fiber-reinforced epoxy matrix composites using ambient- and moderate-temperature curing non-autoclave processes have broad applicability in marine, offshore, and naval applications. This research focuses on the characterization of moisture kinetics of ambient cured carbon/epoxy composites subject to immersion in seawater for up to 72 weeks after prior periods of extended thermal aging. A two-stage model is shown to best describe the overall kinetics and response. The level of maximum moisture uptake shows an increasing trend with the temperature and time of prior thermal aging, reaching asymptotic levels at the highest levels. The transition point is seen to represent a shift between the diffusion and relaxation-/deterioration-based dominant regimes, and the ratio of uptake at the transition point to the maximum uptake can be correlated to the relaxation coefficient. Diffusivity, as expected, generally increases with the temperature of prior aging and shows changes based on the level of post-curing and network changes with time. Moisture uptake kinetics and characteristics developed through the sequence of exposures provide a better understanding of phenomena towards the development of a future comprehensive model capable of long-term prediction based on the sequential prior history of exposure to elevated temperatures and immersion in seawater.

## 1. Introduction

The characteristics of light weight, high specific stiffness and strength, corrosion resistance, potentially high life-cycle durability under harsh marine conditions, good impact resistance, high toughness attributes, tailorability, and relative ease of fabrication in the form of thin skins, stiffened panels, circular cross-section tubes, and sandwich structures make fiber-reinforced polymer (FRP) composites extremely attractive for use in a range of marine applications. Aspects associated with weight reduction and elevated resistance to environmental exposure, which result in reduced maintenance over extended periods of time, lend to the use of FRP to applications such as watercraft, submersibles, and offshore and subsea structures [1]. Beginning with the use of composites for the housing of underwater cables, FRP materials are now used for pressure-resistance housings of profilers, submersibles, and deep-submergence vehicles and in unmanned underwater vehicles [2]. The use of composites continues to grow in applications ranging from small boats and yachts to larger vessels, and there is increasing interest in and demand for carbon fiber-reinforced polymer composites in vessels using advanced designs such as hydrofoils [3,4]. Rubino et al. [1], Moritz et al. [5], Barsotti et al. [6], and Lowde et al. [7] provide extensive reviews of the use of composites for naval vessels, with reviews of the overall area of marine composites being covered in [8,9]. In addition, composites are increasingly considered for applications in which metals are reaching their limits of capabilities [10], which constrains further advances in applications, exploration, and use in areas such as risers [10,11], blades, and equipment for offshore wind and tidal stream devices [1,12]. In these applications and others, the high fatigue resistance of appropriately designed composites and the ability to fabricate lightweight and slender geometric profiles is also advantageous.

Marine and offshore structures are subject to a range of exposures and loads, including those from tides, waves, and storms, with the added effect of seawater on the structure [13]. In addition, these components and structures are often situated in locations that are difficult to access, resulting not only in difficulties in routine maintenance, but also leading to long periods between inspection and maintenance [14]. They often represent mission-critical structural components in marine and offshore applications, including subsea oil and gas platforms, wind and tidal turbines, shipping, coastal structures, and bridges [15,16], and it is therefore important to develop a comprehensive understanding of the long-term response of these materials and components to ensure the appropriate design of structures and components with adequate factors of safety while maintaining economic viability. Incomplete or inadequate knowledge and understanding of materials’ response and durability under in-service conditions limits the increased use of advanced materials in applications in which catastrophic failure could lead to severe damage of the element/structure and/or significant economic losses due to forced periods of non-operation, such as in the oil industry.

The properties of composites depend on the constituents—the fiber and matrix, as well as the bond between them. Though carbon fibers are inert to moisture, both the resin and the fiber–matrix bond are affected by moisture uptake and the resulting reactions with the polymer network. Therefore, it is important to understand the mechanisms of moisture uptake and the effects of moisture on the material and its mechanical performance over time. Solute transport in polymers is related to the availability of molecular-sized holes in the network structure, microcracks and voids, and to polymer–water affinity. Apicella et al. [17,18] proposed three primary modes for sorption: (a) bulk dissolution in the polymer; (b) absorption onto surface holes in the free volume of the glassy network; and (c) bonding between hydrophilic groups on the polymer chain and water molecules. As proposed, these encompass both free and bound water [19,20], thereby resolving the lacuna related to mechanisms based solely on free volume that neglect chemical interactions between water molecules and hydrophilic sites, which intrinsically assume that diffusivity is both concentration and stress dependent. Zhou and Lucas [21] further classified water types in resin as Type I or II depending on the difference in bond complex and activation energy, with the former having lower levels of thermal activation energy, resulting in desorption on reheating. Type I water is related to the disruption of interchain Van der Waals forces, causing increases in chain mobility and resulting in plasticization, whereas Type II bonding results from the formation of hydrogen bonds with the network, resulting in secondary crosslinking between structural segments, which make desorption much more difficult. Desorption levels as a result of these differences in pultruded carbon and glass epoxy composites likely to be used in civil and offshore applications were investigated by Guo et al. [22,23].

Moisture uptake is known to result in both reversible and irreversible mechanisms of deterioration in polymers and composites, ranging from swelling [24], degradation of the molecular structure [25,26], interruption of hydrogen bonds in the polymer network by water molecules [21,27,28], plasticization or increased rigidity depending on the level of saturation [29], increased interfacial degradation [30,31], and changes in mechanical characteristics. Plasticization results in an increase in macromolecular mobility and a decrease in glass transition temperature. The combination of plasticization and swelling can further cause pseudo-ductility [32,33,34], with hydrolysis of the resin resulting in further interfacial degradation and cracking [35], which can also affect out-of-plane strength [36]. This level of damage creates new pathways for the ingress of water [37,38], which can further increase the negative effects of moisture uptake and result in overall performance being constrained by reduced levels of interlayer/interlaminar and interfacial response. Interface degradation can result in the formation of annular microchannels, resulting in more rapid diffusion [39], which is the driver for non-Fickian diffusion phenomena [40]. The extent and type of change at the level of the polymer network and fiber–matrix interphase is not only dependent on the time of exposure but also on the type of aqueous solution [41,42,43,44]. For composites cured under ambient conditions, initial immersion in aqueous solutions can lead to the progression of curing and acceleration in the initial increase of glass transition temperature, followed by significant decreases [44,45,46]. Of special interest to the long-term durability of these materials are the phenomena of hydrolysis and chain scission, which decrease density and can eventually result in the leaching of low-molecular weight species from the bulk polymer and interfacial regions. Effects of the uptake of seawater on the mechanical performance of composites are described in [5,8,9,11,13,14,15,16]. These, are, however, restricted to situations of immersion and humidity only, rather than as a sequence of pre-exposure thermal aging followed by immersion, which would simulate the situations related to extended periods of exposure to heat/fire and high operating temperatures, followed by continued operation in seawater.

The specific effects of seawater are of interest for marine and offshore applications, keeping in mind that aging in sea-air has been noted to have as severe an effect on deterioration as immersion in seawater [47], and thus immersion-based studies could provide indications of long-term durability for applications both in seawater and under worst-case atmospheric aging in humid marine environments [48]. Though there is a substantial body of research on the effects of the immersion of composites in water, differences due to solution type and dissolved chemicals are not well understood, with differences in results even being reported as related to basic aspects such as the maximum uptake and rates of diffusion. Chin et al. [42] reported that moisture uptake in epoxies was Fickian in nature, with the diffusion coefficient in salt solution being almost double that in distilled water. Meanwhile, Scott and Lees [41] reported that neither Fickian nor Langmuir models accurately predict uptake in epoxy when immersed in salt-water solution and distilled water, with apparent diffusion coefficients and maximum uptake levels not varying significantly. This is similar to results reported by Soulier et al., who concluded that diffusion was concentration independent [43]. In contrast, other results [39,49,50,51,52] suggest that salt-water solution results in an increase in diffusivity over that in water with reduced levels of mass uptake at saturation. This is attributed to a reverse osmosis mechanism [49,50,52,53]. Water absorbed from the outside causes an electrolyte to be produced due to the dissolution of internal water-soluble chemicals in the composite. As the water uptake progresses, the electrolyte concentration is diluted, setting up an osmotic pressure. NaCl salts cause the outside solution to be more concentrated than the internally resulting electrolyte, causing reverse osmosis, which in turn causes a decreased uptake of moisture in composites immersed in seawater as opposed to water. The increase in diffusivity can similarly be explained by the enhanced initial formation of cracks and microcavities in the polymer and composite [51]. Though there is a body of work on the effect of seawater exposure, there is still a significant need to gain a comprehensive understanding of these phenomena and the resulting effects [41,44], especially as related to multiple and sequential exposure conditions.

Many applications in the offshore sector include components and structures that are difficult to access and maintain [14], increasing the attractiveness of composites over traditional materials due to their potentially higher durability. These environments often include exposure to high pressure and temperature, with components and equipment required to perform at temperatures between 150 °C and 300 °C [54]. The offshore industry has a history of accidents due to harsh weather and fire, leading to uncontrolled levels of fire and high temperatures for extended periods of time, resulting in severe damage even to the non-fire adjacent areas of the structure. Brkic and Praks [55] provide a summary of incidents, including that of a catamaran-type drilling barge, on which an explosion and fire resulted in gas burning for 13 h; the Enchora central platform, on which a fire lasted a month; the Piper-Alpha platform, where a fire took three weeks to control; and the Adriatic IV, where a fire lasted a week before being brought under control. Though these represent extreme cases, there are other applications that more routinely have components exposed to elevated temperatures for durations between a few hours and a few days [56,57] or are exposed to heat from events such as deflagration during waste transport and storage [58]. In addition, there are temperature requirements for rods [59], risers [11], and tools used in downhole operations at temperatures between 150 and 232 °C [60]. In these and other similar applications, there is a need for assurance of the post-incident operational structural integrity [61,62], and thus the need for a comprehensive understanding of long-term durability under harsh conditions [63] and longer-term exposure to seawater combined with other loading conditions and exposures [64]. With the increasing use of non-autoclave process-based composites in the marine and offshore sectors, there is a need to further study the effects of thermal history and seawater exposure on the long-term integrity and residual characteristics of these materials. Combined with the paucity of data regarding the effects of prior thermal history on the moisture absorption kinetics of non-autoclave cure composites [65] and their increased consideration for use in a seawater environment, there is a need to further investigate the effects of prior thermal aging (as from a fire-based event or high-temperature operating environment) on the longer-term post-event characteristics of carbon/epoxy composites operating in marine and offshore environments.

The current investigation focuses on the effects of seawater immersion on the moisture uptake characteristics of carbon/epoxy composites aged for periods up to 72 h at temperatures up to 232 °C. It builds on the research reported in [62,66], with the aim of better understanding moisture kinetics and the resulting post-exposure moisture uptake characteristics as part of an overall effort to better characterize and predict the long-term durability of these non-autoclave cured, lower cost, carbon/epoxy composites.

## 2. Materials and Methods

### 2.1. Materials and Processes

The carbon fiber-reinforced polymer composite consists of a unidirectional carbon fabric of aerial weight 644 g/m^2^ impregnated with a difunctional bisphenol A/epichlorohydrin-derived liquid epoxy (Epon 828 based) with an epoxide equivalent weight of 185–192 g/cm^3^ and an average molecular weight of about 700, with a polyetheramine-modified polyoxyproylenediamine curing agent with an average molecular weight of about 400. The resulting resin system used in the wet layup process had a viscosity of 600–700 cps at 25 °C and a pot-life of 3–4 h, with a cured density of 1.15 g/cm^3^. Both the fibers and resin were representative of systems already being used in the field in applications similar to those addressed by the current investigation. Epoxy resins are preferred here due to their higher toughness and better long-term performance characteristics under conditions of ambient-temperature curing and subsequent thermal loading. Composite specimens were fabricated with two layers of the carbon fabric using the wet layup process and only manual roller-based pressure without the use of vacuum bags to simulate field conditions. Panels were cured under controlled conditions of 23 °C and 30% relative humidity for seven days, after which there were post-cured for 72 h at 60 °C to enable a uniform level of cure progression. Using acid digestion procedures, the fiber mass fraction was determined to be 60% (i.e., a fiber volume fraction of 49.8%), with a standard deviation of 2–2.5% over all specimens.

### 2.2. Procedure for Conditioning and Moisture Uptake Measurement

Since the investigation was focused only on the determination of moisture kinetics and sorption-related characteristics, moisture uptake was determined through specimens of the nominal size of 25.4 mm × 25.4 mm, following procedures used earlier [66,67]. The thickness of all specimens was equivalent to that formed using two layers of fabric, resulting in a fiber volume fraction of 49.8% with a thickness range between 2.82 mm and 3.24 mm. The variation is due to the manual process used for fabrication to mimic that seen in the field, without external compaction under vacuum.

All specimens were initially conditioned by storing them in a humidity chamber (Electro-tech Systems Controlled Humidity Chamber, Perkasie, PA, USA) at 23 °C and 30% RH for two weeks, after which they were thermally aged at temperatures between 150 °F and 500 °F at intervals of 50 °F, i.e., at 66 °C, 93 °C, 121 °C, 149 °C, 177 °C, 204 °C, and 232 °C, for periods of time ranging from 1 to 72 h. In each case, the specimens were placed in a furnace (Thermo-Fisher Scientific, Waltham, MA) that was already at the specified temperature, then maintained at that temperature ±1 °C for the specified period of time, removed, and then allowed to cool back to room temperature, prior to placing them in seawater for further testing. The moisture uptake specimens were placed in baths filled with seawater maintained at 23 °C ± 1 °C for 72 weeks. The seawater had an average salinity of 33.35, with a dissolved oxygen (DO) concentration of 7.5–8.9 mg/L and a pH of 8.18. The baths were periodically filled to ensure a uniform level and salinity of seawater. Moisture uptake was measured by weighing specimens at periodic intervals. Each specimen was removed using padded tweezers to ensure minimal pressure and no contamination by human hands, patted dry with tissue paper, and weighed, after which they were reinserted into the baths. The time period for the operation was standardized to ensure consistency and to avoid effects of varying levels of evaporation through the specimens. To ensure as little change to surface conditions as possible, specimens were not rubbed, since this could remove salts that were collected on the surface, the removal of which could affect further uptake progression. A minimum of three specimens were tested for each combination of time and temperature of thermal aging and each period of immersion in seawater. Percentage mass change, i.e., moisture uptake, was determined as:

(1) $$M=\frac{M_{t}-M_{0}}{M_{0}}\times 100$$

where *M_t_* is the mass of the specimen after immersion for time *t*, and *M*_0_ is the original mass prior to initiation of immersion.

## 3. Results and Discussion

**Figure 1:**
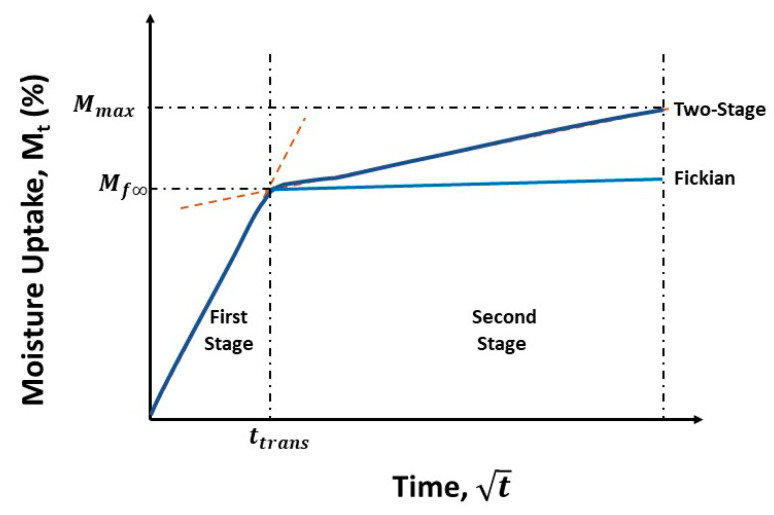
Schematic of moisture uptake curves following different models.

Moisture uptake in polymers and composites is often described in terms of a diffusion-dominated process that assumes no swelling due to water uptake and does not account for stress dependent diffusion. The Fickian model, in which uptake is characterized by two parameters, i.e., diffusivity, *D*, and uptake level at saturation, $M_{f\infty }$, can be expressed following [68] as:

(2) $$\frac{M_{t}}{M_{f\infty }}=1-exp\left[-7.3{\left(\frac{Dt}{h^{2}}\right)}^{0.75}\right]$$

where *M_t_* is the uptake measured at time, *t*, and *h* is the thickness of the specimen. Given the assumed linearity of the initial uptake in the Fickian representation, the diffusivity can be obtained from the initial slope of the uptake versus the $\sqrt{t}$ curve (Figure 1) as:

(3) $$D=\pi \left(\frac{h}{4M_{f\infty }}\right){\left(\frac{M_{2}-M_{1}}{\sqrt{t_{2}}-\sqrt{t_{1}}\;}\right)}^{2}$$

where *M*_2_ and *M*_1_ are uptake levels at times *t*_2_ and *t*_1_, respectively. Small-molecule diffusion in polymers and composites has been noted to show significant deviation from a simple Fickian response due to aspects such as moisture-induced damage, especially at the fiber–matrix interphase level, adsorption-related phenomena, and relaxation, all of which are slower than the initial diffusion process. In the current investigation, these phenomena lead to uptake continuing to increase over time rather than attaining the asymptotic Fickian saturation level (Figure 1). This combination of concentration gradient-driven Fickian diffusion and other slower time-dependent processes is thus better characterized by a two-stage model, in which the differential rate for the slower, relaxation- and moisture uptake-induced damage processes can be described following Behrens and Hopfenberg [69] as:

(4) $$\frac{dM_{t}}{dt}=k\left(M_{\infty R}-M_{tR}\right)$$

where *k* is a coefficient representative of relaxation- and moisture uptake-related damage, and $M_{\infty R}$ is the level of moisture uptake due to these phenomena. In line with Bagley and Long [70], and following the initial work of Bao et al. [71], and as applied to composites [68,72], the two-stage process can be expressed as:

(5) $$\frac{M_{t}}{M_{trans}}=\left(1+k\sqrt{t}\right)\left\{1-exp\left[-7.3{\left(\frac{Dt}{h^{2}}\right)}^{0.75}\right]\right\}$$

where *M_trans_* is the moisture uptake level representative of the transition between the two stages and is equivalent to $M_{f\infty }$ in Figure 1. When *k* = 0, Equation (5) reverts to the Fickian form of diffusion modeled by Shen and Springer [68] in Equation (2). It is thus of interest to study the effects of prior thermal aging not just on the parameters of diffusion, *D*, and relaxation/deterioration, *k*, but also on the different uptake levels and the transition point between the two stages, since these as a set can more comprehensively describe the interacting phenomena and the effects at play under such conditions of exposure.

**Figure 2:**
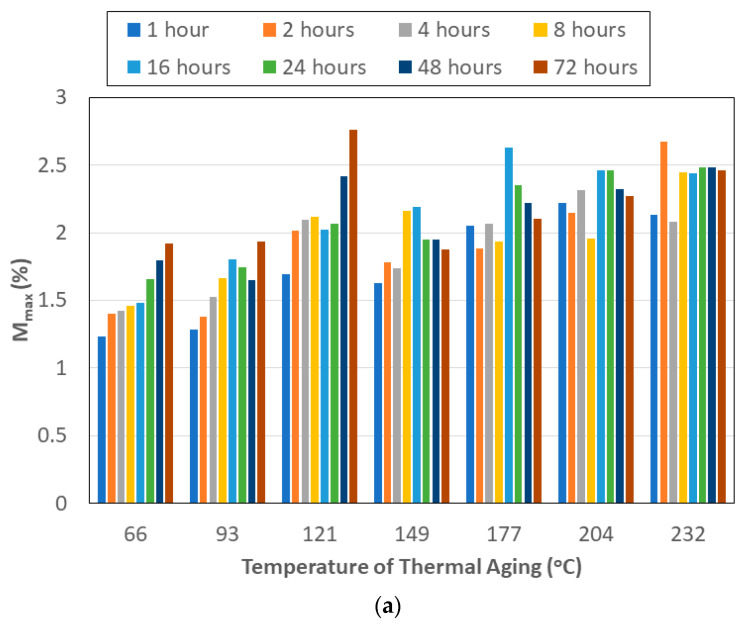

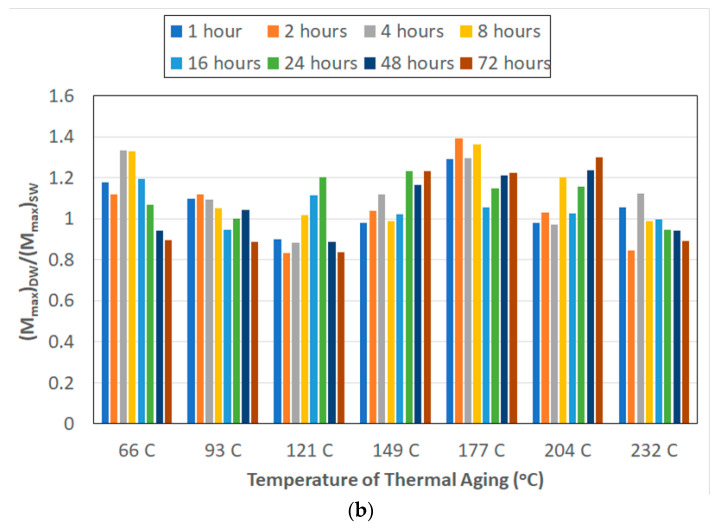
(**a**) Level of maximum seawater uptake as a function of temperature and time of prior thermal aging. (**b**) Ratio of maximum uptake from immersion in deionized water to that in seawater as a function of time and temperature of prior thermal aging.

The rate of uptake in a composite is generally seen to increase with an increase in the temperature of exposure [30,40]. In the current case, however, thermal aging occurs prior to immersion in the solute, seawater, and hence the sequence of exposures and prior thermal history are expected to play a role, especially in light of the competition between temperature and moisture-induced post-cure of the polymer, the change in network and interface structure due to thermal aging, and degradation processes emanating from immersion in seawater. As can be seen in Figure 2a, the level of maximum moisture uptake, *M_max_*, shows an increasing trend with the time of prior aging between 66 °C and 121 °C. The decrease in uptake noted for specimens subject to 16 h of pre-aging at higher temperatures is in line with earlier results reported by Nogueira et al. [28] of the decrease in uptake with the increase in the degree of cure due to the higher resulting crosslink density of the network, which decreases the presence of molecular-sized holes in the polymer structure. This results in lower free volume, and hence a decrease in the level of moisture uptake. At the two highest levels of thermal aging of 204 °C and 232 °C, the change in the uptake level between 48 h and 72 h was less than 2%, well within the scatter that could be expected from such measurement. The average uptake level at each temperature across all times of exposure increases with the temperature of pre-aging, with the lowest being 1.54% at 66 °C and the highest being 2.4% at 232 °C, which follows the trend indicated in earlier studies of immersion in solutions at increasing temperatures [73]. In comparison, the average across all temperatures of prior thermal aging for each level of thermal exposure increases from 1.75% at 1 h to 2.19% at 72 h, with the levels remaining fairly constant from 16 h onwards, indicating the decreasing dominance of the time of exposure after a threshold as related to changes that would result in increased uptake. The existence of a threshold is in line with the results of Loos and Springer [40] of the maximum uptake being less sensitive to temperature, which can further be explained through the coupling between heat transfer and moisture diffusion. In the case of the current investigation, the increasing trend of uptake with the temperature of prior thermal aging is in line with results reported by Morgan et al. [74] of moisture sorption being enhanced by thermal spikes that cause changes in the polymer network structure, resulting in additional free volume that was previously inaccessible to water molecules. The threshold of 16 h corresponds to the level at which post-curing was estimated to reach a maximum threshold in an earlier study [66].

**Figure 3:**
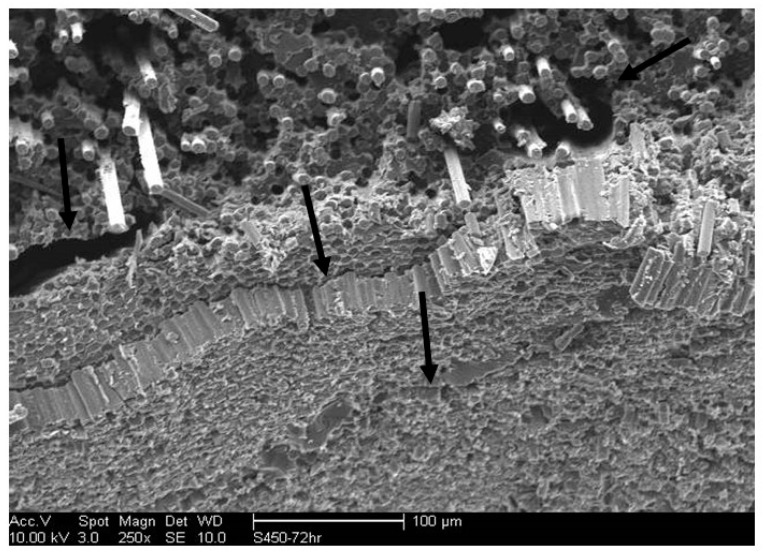
Debonding and separation between fiber layers after immersion in seawater (Arrows indicate areas of separation of fiber layers).

Uptake in seawater has been reported previously to be less than that in water [42,43,49,50,51], which is largely confirmed by results from this investigation through comparison with results on the same materials system immersed in deionized water [66]. Figure 2b shows that for most cases, the ratio of the maximum uptake in deionized water is greater than that in seawater, i.e., (M_max_)_DW_/(M_max_)_SW_ > 1, where the subscripts DW and SW signify post-thermal aging immersion in deionized water and seawater, respectively. The major deviations from this trend occur at the longest periods of aging (48 and 72 h) at 66 °C, 93 °C, 121 °C, and 232 °C. Overall, however, there are no clear trends between the two solutes, indicating the complex interaction and phenomena, including those of reverse osmosis due to salt concentration of the surfaces creating a straining effect that also decreases uptake in the second stage. At 232 °C, the deterioration in the resin is, however, high enough to cause separation between layers of fibers (Figure 3), resulting in a significant increase in wicking, which results in the higher uptake in seawater over that in deionized water at these levels. Tam et al. [75] similarly reported earlier that a salt solution caused the maximum loss in the interfacial bond due to degradation at the resin and fiber–matrix interface levels. The average ratio of uptake over all temperature levels for the same period of thermal aging ranges from a minimum of 1.06 at 16 h to a maximum of 1.16 at 8 h, whereas that due to the overall period of exposure at different temperatures of aging ranges from a minimum of 0.96 at 121 °C to a maximum of 1.25 at 204 °C, indicating the complex interaction between the temperature and time of prior thermal aging and the effects of seawater immersion. The latter condition itself leads to a complex set of mechanisms, which have only recently started to be characterized as related to long-term effects [76].

**Figure 4:**
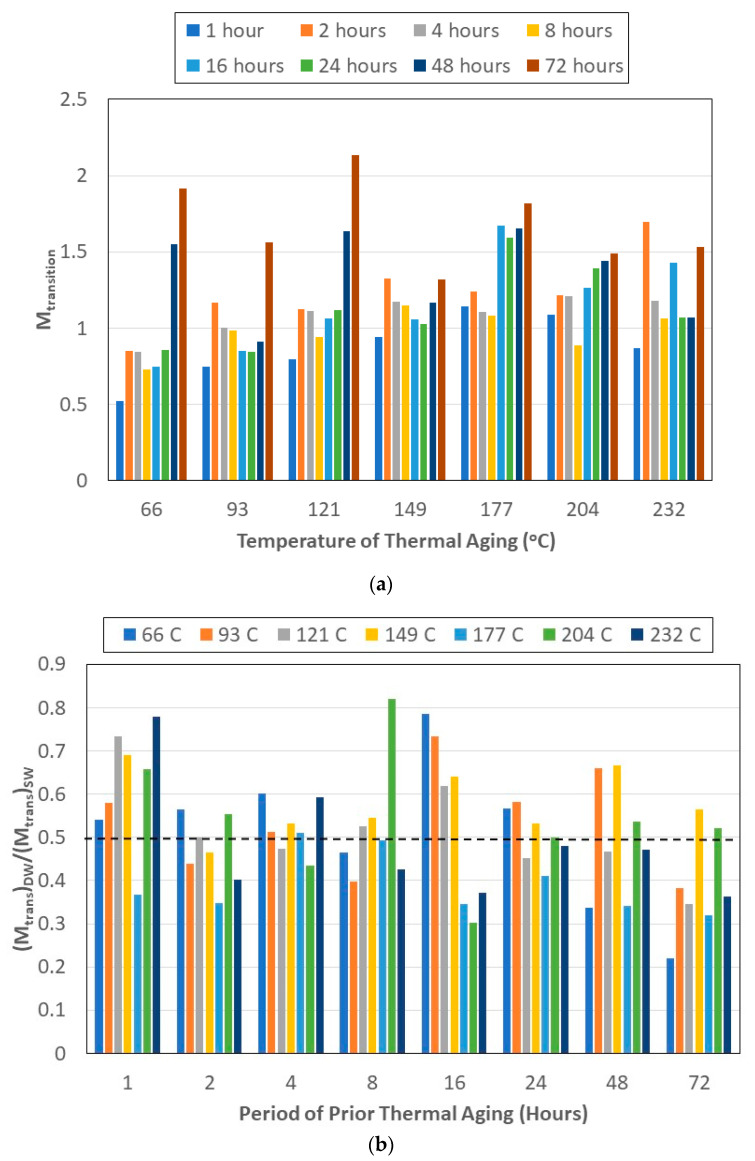
(**a**) Level of uptake at the transition point as a function of temperature and time of prior thermal aging. (**b**) Ratio of transition level uptake from immersion in deionized water to that in seawater as a function of time and temperature of prior thermal aging.

The transition point between the diffusion and relaxation-/deterioration-dominated regimes is an important parameter, and the levels of uptake at this point are shown in Figure 4a. For all temperatures of prior thermal aging, the uptake is seen to increase from the 1 h to 2 h levels and then decrease till the 8 h level, presumably as an initial effect of post-curing, after which it either continued to decrease further (till 24 h at 93 °C and 149 °C) or increased again, with the highest uptake being at the longest period of thermal aging for all temperatures except 232 °C. Though desorption mechanisms and extent were not assessed, it is important to note that the transition threshold also signifies the change from the dominance of mechanisms related to free and bound water, since the second stage effects are related to relaxation phenomena, which are largely due to the latter. The uptake of water not only causes changes in the polymer network but also increases stresses at the interface, resulting in greater fiber–matrix contact and decreased wicking through those spots. However, as the uptake increases, the deterioration increases and causes wicking paths to not only reopen but also increase. In addition, hydrolysis reactions in the bulk resin can also result in this increase following a drop. Thermal aging at 232 °C was noted earlier [62] to result in a complex state of deterioration and a fairly rapid drop in glass transition temperature, as determined through dynamic scanning calorimetry (DSC) after 4 h of aging. Resin-dominated mechanical characteristics demonstrated a range of deteriorative mechanisms, confirming a complex thermally induced state, which results in the variation in uptake, as seen based on fairly localized damage. The levels of maximum uptake are generally higher in deionized water than in seawater. This is depicted in Figure 4b using the ratio (*M_trans_*)*_DW_*/(*M_trans_*)*_SW_*, where *M_trans_* represents the uptake level at the point of transition. Thus, as a result of immersion in seawater, the lower the value, the greater the uptake. The level of moisture uptake of the transition point is significantly higher (about twice on average) in seawater than in deionized water. The relatively higher level of uptake in the transition point can be related to earlier reported results [42,49] of significantly higher diffusivity in saltwater/saline solutions. Kahraman and Al-Harthi [49] attributed this to the increased formation of microcavities in the bulk epoxy. In general, the difference increases with the time of exposure, with the highest ratios being seen from 24 h of prior aging onwards, irrespective of the temperature of thermal aging.

**Figure 5:**
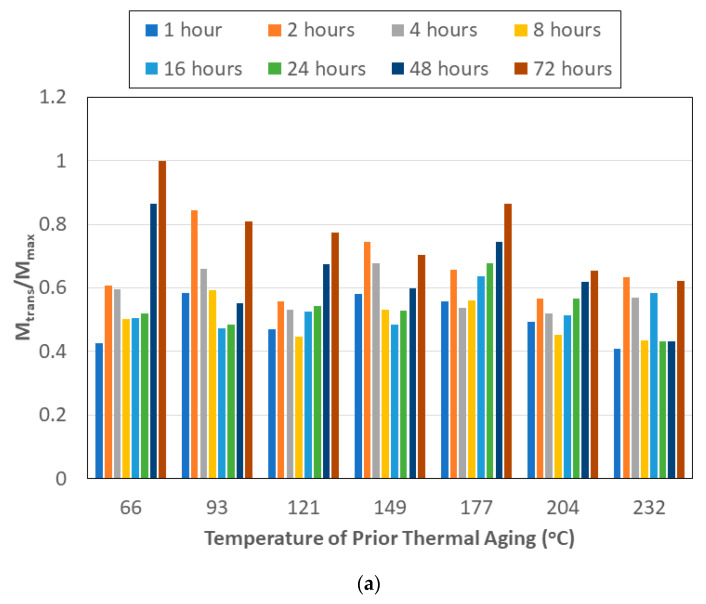

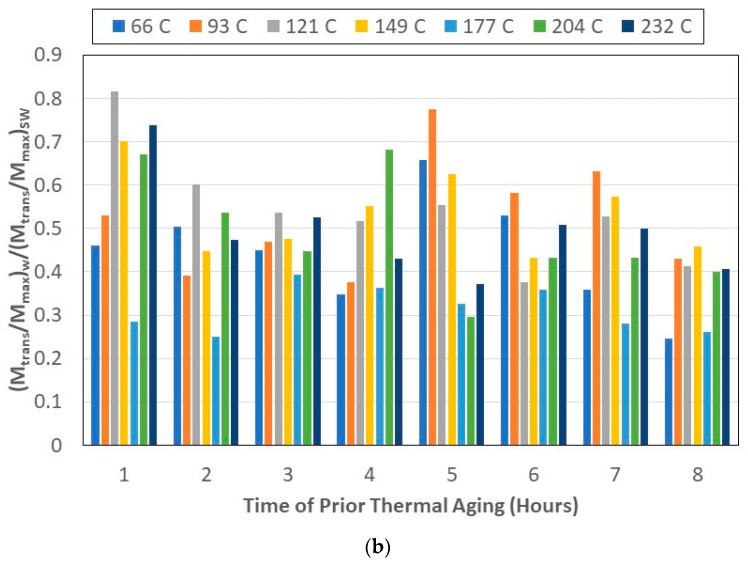
(**a**) Ratio of uptake at the transition point to the maximum uptake level as a function of temperature and time of prior aging. (**b**) Ratio of uptake levels at transition and maximum due to immersion in deionized water to that in seawater as a function of time and temperature of prior thermal aging.

Though the magnitudes of moisture uptake corresponding to the transition and maximum levels are of interest, the ratio of these two levels, *M_trans_*/*M_max_*, also provides insight into the relative influence and length of the two primary mechanisms involved—an initial diffusion-dominated regime and a subsequent relaxation-/deterioration-based regime that has a far more complex set of competing phenomena, including that of longer-term hydrolysis of the resin, irreversible changes in the network including those of post-curing, and of deterioration through the growth of microcavities and increased fiber–matrix debonding. As seen in Figure 5a, there is an initial increase in the ratio, indicative of an increased uptake in the diffusion-dominated regime, followed by a drop that extends between 8–16 h of prior thermal aging, followed by an increase again at all temperatures of prior aging except the highest of 232 °C. These trends are in accord with the phenomena reported in earlier studies related to moisture absorption and temperature [77]. It is noted that the first stage of moisture uptake can be expected to end later, as the degree of polymerization attained in the polymer and composite prior to immersion is higher. Simultaneously, it must be emphasized that though this increases the free volume, the level of energy needed for the water molecules to diffuse across the network and fill the free volume also increases [78], which can be seen through the decrease in ratio followed by the increase at longer periods of exposure, indicating that past a threshold level, the effects of relaxation/deterioration on moisture uptake increase. With the exception of thermal pre-aging at 232 °C, the ratio of *M_trans_*/*M_max_* increases from 16 h onwards, with the increase initiating even earlier at the 8 h level for the specimens pre-aged at 66, 121, 177, and 204 °C. For each temperature of thermal aging, the highest resulting value of *M_trans_*/*M_max_*, i.e., the highest relative uptake at the transition point, is due to the longest period of prior aging of 72 h. In Figure 5b, a comparison of trends in the ratio of uptake parameters through immersion in deionized water and seawater after thermal aging shows an absence of dominant trends, again emphasizing the complexity of mechanisms at play and the need for further study of interactions due to exposure conditions.

**Figure 6:**
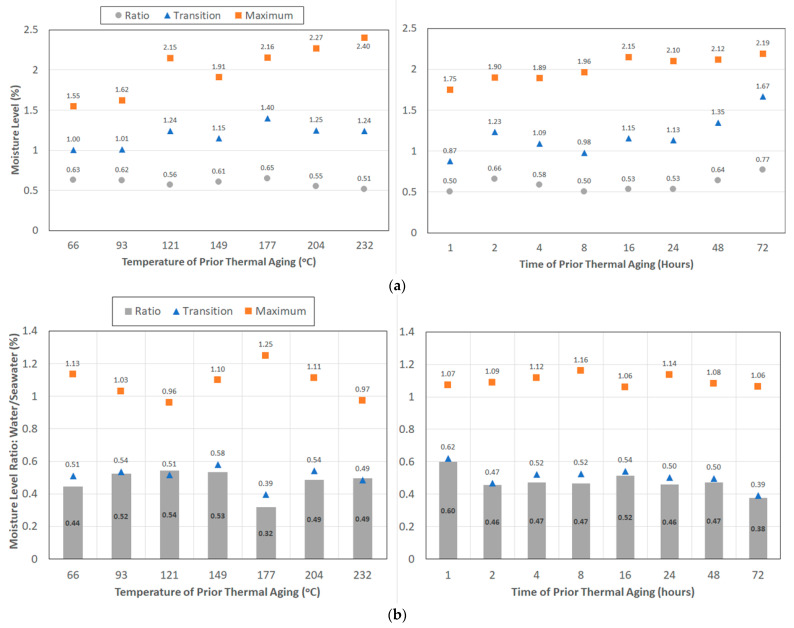
(**a**) Condition-averaged values for moisture uptake levels due to immersion in seawater. (**b**) Condition-averaged values for the ratio of level attained in water to those in seawater.

Though there are a wide range of phenomena and interactions due to effects of the temperature and time of prior thermal aging and immersion in solution, on the resulting uptake curve, it is beneficial to understand the relative effect of the two factors of prior aging—the temperature and period of exposure—on uptake response. Following the averaging method used by Baghad et al. [79], which was used previously to assess effects after immersion in deionized water [66], Figure 6a,b depict the results for uptake levels due to immersion in seawater and a comparison between immersion in water and seawater (expressed as a ratio), respectively, in terms of the three moisture uptake characteristics—transition, maximum, and ratio—considered in the study by averaging across the ranges in each case. As seen in Figure 6a, the level of maximum uptake increases with both the temperature and time of thermal pre-aging, but the rate and level of increase is substantially greater due to a change in the temperature of prior thermal exposure. Though the maximum level of seawater uptake increases almost linearly from 149 °C to 232 °C at a rate of 0.006% per °C increase in temperature, it attains a near asymptotic level between 2.1 and 2.19% moisture uptake after 16 h of exposure across all temperatures of prior thermal aging, indicating the presence of a threshold. In this case, as mentioned earlier, this correlated to the attainment of the maximum glass transition temperature, which is significant from a design and post-extreme event assessment perspective [80]. In comparison, the transition level is affected to a significantly greater amount by the time of exposure, both in absolute levels of moisture uptake and the relative increase with time, with the increase over the range considered (i.e., 1–72 h) being 92%, whereas the increase across the temperature range (66 °C–232 °C) is only 40%. The greater effect of the time of pre-exposure over Stage I of the uptake curve reflects the effect of time at a constant temperature on the diffusion-dominated regime, as would be expected from a Fickian response that is time dependent. As discussed previously, the ratio *M_trans_*/*M_max_* is of special interest in the assessment of mechanisms and the relative roles of diffusion and deterioration/relaxation. As can be expected from the results related to levels of *M_trans_*, the time of pre-exposure has a greater overall effect, especially at longer periods of thermal pre-aging, with the increase being pronounced from 24 h onward following a fairly constant level between 8 and 24 h of thermal aging across all temperatures.

A comparison of effects due to immersion in deionized water with those from seawater for the same uptake characteristics is shown in Figure 6b. It is of interest to note the very narrow band of response in terms of the maximum uptake ratio as a function of time of exposure, indicating that though levels are different in the two solutions, the effect of time on mechanisms and rates is similar. A limited similarity is also seen for the transition level between 4 h and 48 h of thermal aging prior to immersion. Though significant further investigations need to be conducted to enable a greater understanding of the similarities and differences, it does appear that time-based mechanisms may cause uptake to asymptotic levels after the attainment of thresholds, providing a direction for further research into understanding the differences in moisture kinetics resulting from immersion in different solutions.

**Figure 7:**
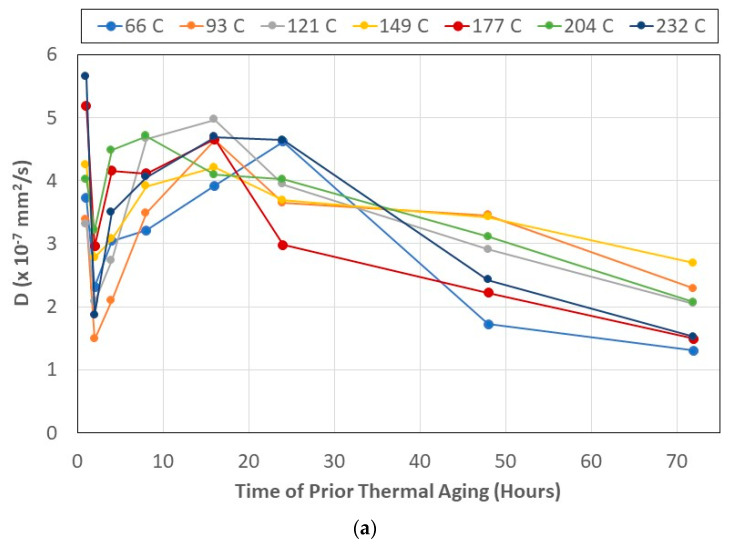

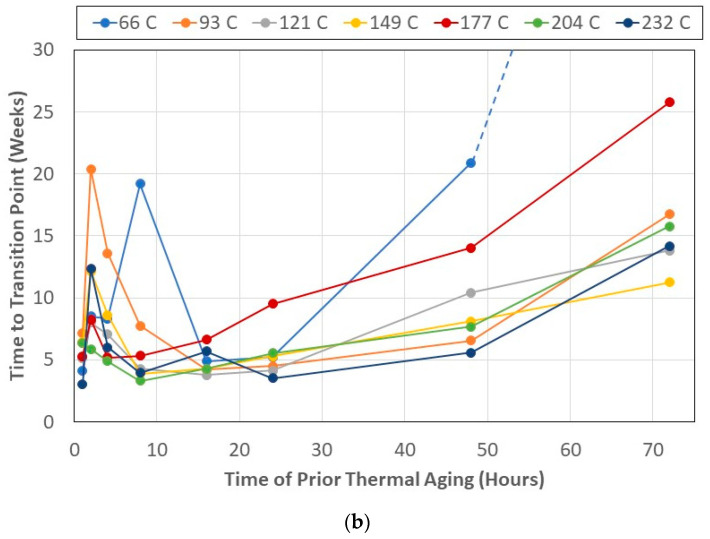
(**a**) Diffusivity in the two-stage model for immersion in seawater as a function of time and temperature of prior thermal aging. (**b**) Time taken to attain the transition level of moisture uptake due to immersion in seawater as a function of time and temperature of prior thermal aging (the trend for 66 °C beyond 48 h appears anomalous and is hence dashed).

In the two-stage process of moisture uptake, the first stage, extending up to the period of transition, is diffusion dominated. Considering the earlier discussion on the effect of the time of prior thermal aging on the moisture uptake levels at the transition point, it is of interest to assess diffusivity as a function of the time of previous thermal exposure for different temperatures of thermal aging. In the case of thermally activated processes such as those due to immersion in solution, an increase in the temperature of the solution is generally seen to result in an acceleration of diffusion [68,72]. In the current case, however, the two drivers—prior thermal aging and moisture uptake—cause different and often opposing effects, resulting in complex interactions at the level of physical aging, temperature-driven post-curing, network structure changes, and even microcracking in the bulk resin and increased fiber–matrix debonding, as well as uptake-driven plasticization. The ratio of free and bound water also changes, increasing the coupling between physical aging and plasticization [32]. In the current investigation, these interactions are even more complex with increasing uptake, to an extent reversing the effects of prior thermal aging on the polymer and composite. As shown in Figure 7a, diffusivity, *D* in Equation (5), initially decreases and then increases to a peak that is attained for specimens exposed to 16 h of thermal aging at all temperatures except at 66 °C (at which the peak occurs for specimens exposed to 24 h of aging) and at 204 °C (at which the peak occurs for specimens previously exposed to 8 h of thermal aging), after which it decreases till the 72 h level. The general trends of an increase followed by a gradual decrease are in line with the phenomena of initial changes in the polymer network that result in an increasing progression of curing, causing an increase in diffusivity, followed by longer term structural swelling and changes causing a subsequent reduction in diffusivity, i.e., the rate of uptake as the response reaches the transition stage. It is thus of interest to assess the time taken to reach the transition stage in the moisture uptake response as a function of the time of prior thermal aging, as shown in Figure 7b.

An initial peak is reached within two weeks for all temperatures except 66 °C, at which point it takes a significantly longer period of eight weeks. As the period of prior thermal aging is increased, the time to reach the transition level of moisture uptake decreases for all temperatures of thermal aging and then increases again, indicative of the initial rapid diffusion along the fiber–matrix interface and through free volume, followed by slower motion through an evolving and swelling polymer network undergoing largely reversible plasticization, and then a faster increase as further wicking occurs along a deteriorating fiber–matrix interface and through microcavities. These mechanisms extend the time to attain the transition point, representing the end of the diffusion-dominated first stage of uptake. As can be expected, there is a generally linear relationship between diffusivity, D, and the time to attain the transition uptake level, with diffusivity decreasing with the increase in the time taken for moisture uptake. The exception to the largely linear trend is for specimens pre-aged by exposure to 66 °C, wherein the trend is still one of decrease but staged with changing rates through the set. The overall rate of decrease is the fastest at the highest temperature of 232 °C, with rates at other temperatures as listed in Table 1.

The two highest rates are at 66 °C and 232 °C, with the rest varying, without a clear trend in the rate with the change in temperature. This is due to the multiple mechanisms at play even in the first stage of moisture uptake based on prior thermal history, which leads to competition between mechanisms of post-curing and deterioration at the bulk polymer and fiber–matrix levels [62,66,80]. The current condition of immersion in seawater for an extended period of time (72 weeks) following a range of pre-immersion thermal aging regimes thus adds further coupling and interaction of phenomena other than that investigated in the comprehensive study of the coupling between plasticization and physical aging in an epoxy in a wet environment [32] and that of water content on physical aging reported earlier by Zheng et al. [81].

**Figure 8:**
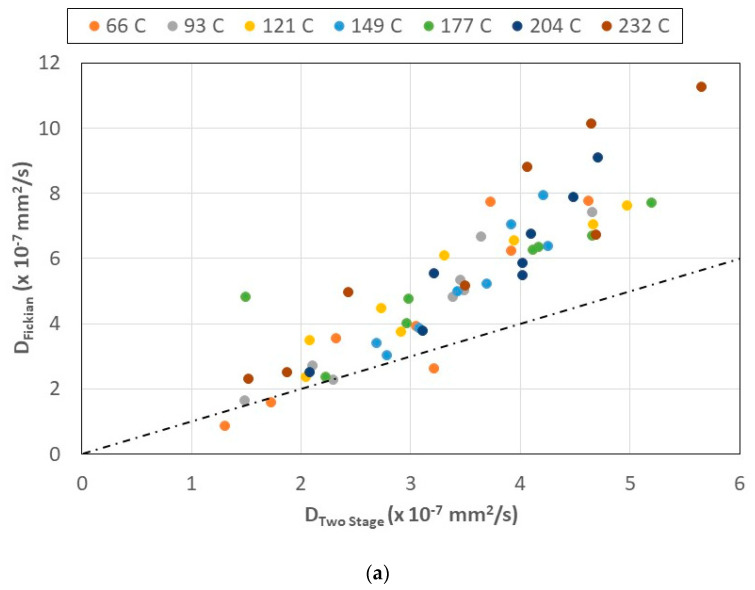

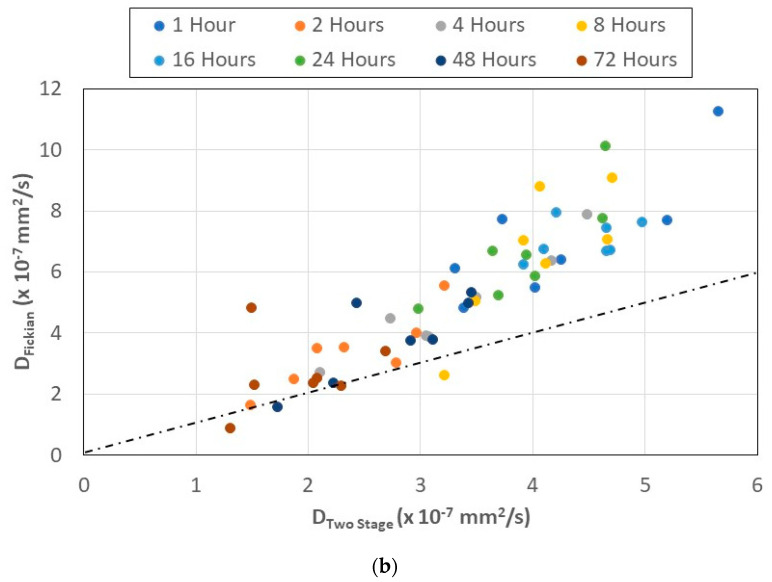
(**a**) Comparison of representation of diffusivity as a function of temperature of prior thermal aging (the dashed line represents a ratio of 1). (**b**) Comparison of representation of diffusivity as a function of time of prior thermal aging (the dashed line represents a ratio of 1).

The two-stage diffusion model represented by Equation (5) assumes that structural relaxation, irreversible deterioration, and structural network changes occur over a much longer time scale than diffusion, whereas the Fickian model assumes a much more rapid uptake as a distinct function of $\sqrt{t}$, as in Equation (3). Desorption and re-absorption experiments indicate irreversible network structural changes in diffusion characteristics, which are history dependent [72,73]. This is further borne out by other tests reported on the effect of thermal spikes and prior thermal history on the effect of moisture uptake during the hydrothermal testing of composites [82,83,84]. It is thus of interest to compare the diffusion coefficients obtained through the use of the two different models, Fickian and two-staged, using the value of *M_trans_* (=*M_f∞_* in Equations (2) and (3)) as the asymptotic saturation value for the Fickian model. This transition between the diffusion- and relaxation-controlled regimes can be considered as a quasi-equilibrium uptake level representative of the uptake largely due to diffusion, as suggested by Bao and Yee [85]. Figure 8a,b plot the two coefficients as a function of the temperature and time period of prior thermal aging, respectively, with the values of slopes (which indicate levels of correlation) and R^2^ values being reported in Table 2.

As would be expected, the Fickian values are higher, since they assume a single mechanism with a rapid attainment of saturation, whereas in the two-stage model, although diffusion is the dominant mechanism, other mechanisms are also assumed to be present. Further, it should be emphasized that the Fickian model effectively treats composites as homogenized and assumes no swelling as a result of moisture uptake, as well as that the process is diffusion dominated, with diffusion being significantly faster than the directly and indirectly caused processes of relaxation [69,86,87]. As a result of this, the model does not describe, nor account for, moisture-induced swelling, volumetric change, or the later stress-dependent uptake. From a mechanistic perspective, this difference is critical, since the swelling stress resulting from the initial absorption of water causes the network structure to expand, and thus creates additional space for free water uptake, which in turn causes a decrease in the glass transition temperature [88,89]. However, a single-stage model would predict a continuous drop in T_g_ with an increase in moisture content rather than the attainment of a threshold, as reported [73]. The slope is the highest at the two highest temperatures of pre-aging, 204 and 232 °C, with the specimens that were pre-aged at lower temperatures showing slopes that are fairly constant (between 1.5 and 1.56), indicating similar interactions between diffusion and longer-term relaxation initiation at the lower temperatures. In comparison, no distinct trend is noted with time of pre-aging, although the lowest ratios of the coefficients (i.e., slope) are noted at the two longest periods of aging, indicating the increased dominance of diffusion-based processes at the longer periods of time. This can be related to increased deterioration of the fiber–matrix interfaces, resulting in greater uptake during the first stage.

**Figure 9:**
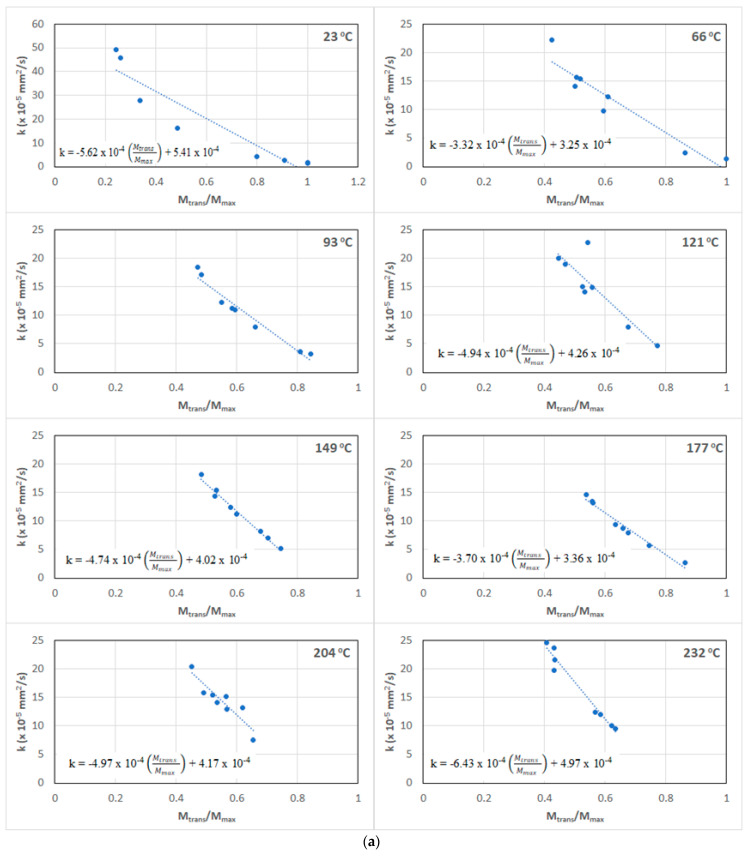

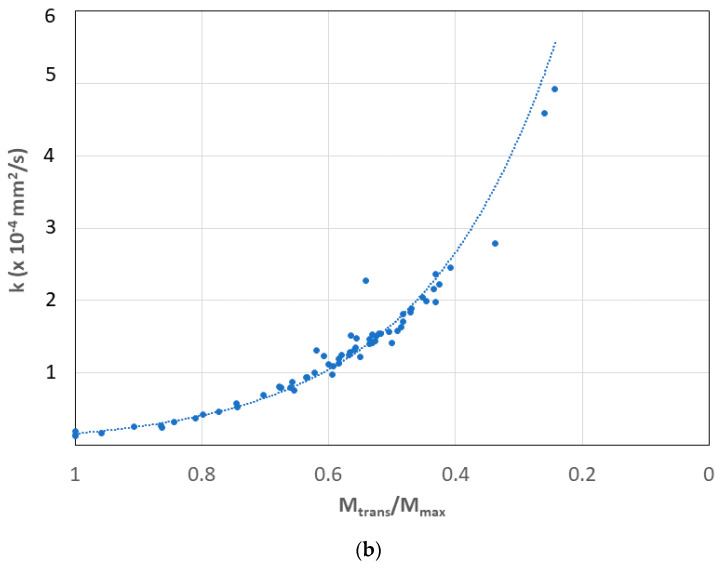
(**a**) Relaxation/deterioration coefficient as a function of *M_trans_*/*M_max_* for different temperatures of prior thermal aging. (**b**) Exponential relation between *k* and *M_trans_*/*M_max_* across the full range of temperatures and times of prior thermal aging.

The lacuna in the Fickian model, described above, is addressed in the current investigation by the inclusion of the relaxation/deterioration mechanisms through *k*, as in Equations (4) and (5). This intrinsically supports both longer-term relaxation and deteriorative phenomena and allows for the inclusion of multiple diffusion mechanisms involving free and bound water at different stages of the uptake history, including in the interfacial region and in cracks and voids, as hypothesized by Bone et al. [14]. The mechanisms of uptake are driven by relaxation, network changes, and overall deterioration with increasing degradation of the fiber–matrix interface and debonding, in addition to microcracking, hydrolysis, and swelling stress-induced microcracking, which takes place as moisture uptake increases. In the case of hygrothermal exposure, these effects could be expected to be functions of the temperature of the solution, with an increase in temperature accelerating structural relaxation and consequently increasing the rate of uptake in the second stage. However, in the current investigation, significant levels of irreversible change, including that of increased cross-linking, occur during the process of thermal pre-aging, resulting in complex interactions with the moisture absorbed because of subsequent immersion in the solution. The uptake level of *M_trans_* represents a useful metric to assess changes, especially in the form of the uptake ratio. The ratio *M_trans_*/*M_max_* effectively relates to the relative range of each of the two stages, with the second stage being more dominant as the value of *M_trans_*/*M_max_* decreases, i.e., as the transition occurs earlier in the uptake curve. Figure 9 shows plots of the relaxation/deterioration coefficient, *k*, as a function of the moisture uptake ratio, *M_trans_*/*M_max_*, for the range of temperatures at which pre-immersion thermal aging was conducted. To enable comparison with the aging process under ambient conditions, a set of specimens aged in controlled conditions at 23 °C is also included. Figure 9a shows the change in the relaxation coefficient, *k*, as a function of *M_trans_*/*M_max_* at each temperature of prior thermal aging, with a linear relationship described by:

(6) $$k=m\;\left(\frac{M_{trans}}{M_{max}}\right)+k_{0}$$

where *m* is the slope and reflects the rate of change in the relaxation/deterioration coefficient with a decrease in the Stage II portion of the uptake curve, i.e., *k* decreases, as expected, as Stage I occupies a greater percentage of the overall uptake. The highest rate of change is seen at the highest temperature of thermal aging, with the lowest (i.e., the slowest change) being at the two lowest temperatures of 66 °C and 93 °C. For specimens at 23 °C, at which there is no elevated temperature induced post-cure, the rate of deterioration in *k* is higher than that of all specimens except at the highest temperature of pre-exposure. Post-curing results in a reduction in potential relaxation processes after immersion in seawater at all except the highest temperature, at which damage to the fiber–matrix interface level and in the bulk polymer itself results in rapid changes in *k* as the length of Stage II uptake increases. The relative similarity of rates between 121 °C and 204 °C reinforces the observations discussed earlier as related to moisture uptake characteristics within this range.

Relaxation processes are often described through exponential relationships of the type:

(7) $$G\left(t\right)=fn\;\left\{\exp\left(\frac{-t}{\tau }\right)\right\}$$

where *t* and *t* are time and characteristic time, respectively. In the current case, the key parameters are related to the levels of moisture uptake rather than directly to time, and it is hence of interest to see whether the relaxation parameter for the range of conditions tested could be generally described through an exponential relation. Figure 9b depicts the set of data of relaxation coefficients, *k*, plotted as a function of *M_trans_*/*M_max_*, and it can be seen that the data fit an exponential curve:

(8) $$k=0.0017\;e^{-4.6702\left(\frac{M_{trans}}{M_{max}}\right)}$$

with a R^2^ values of 0.96 when ambient (i.e., 23 °C) data are included, and

(9) $$k=0.0018\;e^{-4.745\left(\frac{M_{trans}}{M_{max}}\right)}$$

with an R^2^ square of 0.92 when only the thermally pre-aged sets are included. The relatively slight difference in parameters suggests that a single master curve could be used to predict the response, and that if parameters for the material were known, one could determine the moisture history through its uptake behavior as modeled. The use of the ratio *M_trans_*/*M_max_* is necessitated, since relaxation is not just a function of moisture uptake-driven mechanisms but rather depends on the evolving network structure of the bulk polymer and the composite and the integrity of the fiber–matrix interface, which is intrinsically dependent on the prior temperature and time of thermal aging. The relaxation/deterioration coefficient, *k*, increases as the ratio *M_trans_/M_max_* decreases, i.e., as the uptake in Stage II becomes larger, i.e., the diffusion-dominated first stage ends earlier in the uptake process. Thus, as noted, though a direct relation between *k* and time does not exist, a relation with the ratio *M_trans_*/*M_max_* can be determined, as seen in Figure 9a,b, emphasizing the complex interactions between the time and temperature of prior thermal aging and subsequent immersion in seawater.

**Figure 10:**
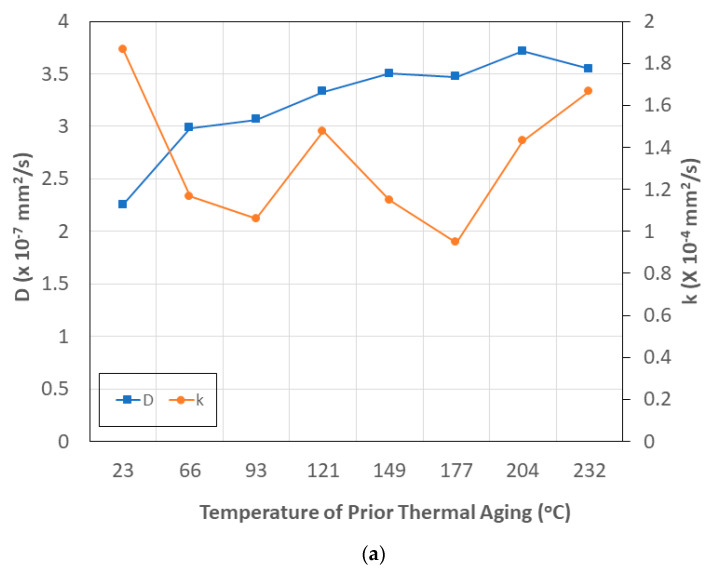

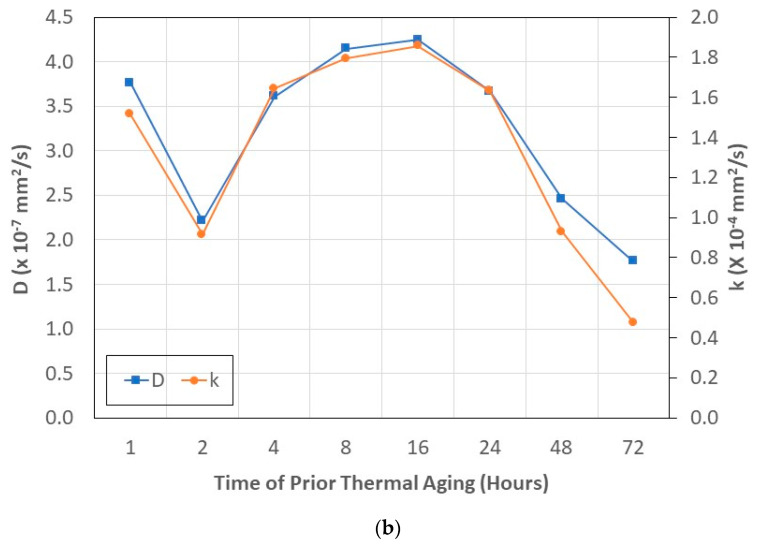
(**a**) Condition-averaged values for diffusion, *D*, and relaxation/deterioration, *k*, coefficients due to immersion in seawater as a function of temperature of prior thermal aging. (**b**) Condition-averaged values for diffusion, *D*, and relaxation/deterioration, *k*, coefficients due to immersion in seawater as a function of time of prior thermal aging.

Given the importance of the two coefficients, *D* and *k*, in the formulation of the two-staged model, it is of interest to understand the relative effects of the temperature and time of prior aging on both. Figure 10a,b show the results of averaging across the range of values of one variable on the other, in terms of temperature and time of pre-aging, respectively. As can be seen from Figure 10a, diffusivity, *D*, increases with the temperature of thermal pre-aging, approaching an asymptote at the three highest temperatures, whereas the relaxation/deterioration coefficient, *k*, shows a varying trend with an increase at the highest temperatures. The former is in line with the effect of increasing the temperature of the solution on diffusion, suggesting that the effect of a sequential exposure is similar in nature, whereas the latter suggests complex interactions between free and bound water effects and those of thermal and moisture kinetics, as discussed previously. As seen in Figure 10b, both coefficients show similar response trends as a function of the increase in the time of prior thermal aging over the full temperature range, with the initial decrease followed by an increase to the previously identified level at which the peak T_g_ through DSC was measured, and then a decrease in a mode similar to that discussed earlier in the section for the maximum level of uptake.

## 4. Summary and Conclusions

Results from moisture uptake tests conducted over a 72-week period of immersion in seawater on specimens previously thermally aged at temperatures between 66 °C and 232 °C for periods of time between 1 h and 72 h were analyzed using a two-stage diffusion model that incorporated both diffusion and relaxation/deterioration phases, transitioning from one dominant mechanism to the other to gain a better understanding of moisture kinetics and related mechanisms. The following conclusions can be drawn from the investigation:

(1) The two-stage model better describes the moisture uptake and provides a wealth of moisture kinetics information, not only as related to stages and rates of uptake but also in terms of diffusion and relaxation/deterioration coefficients, representing the complex interactions between mechanisms initiated by the extent of prior thermal aging and those subsequently initiated through immersion in seawater.
(2) The level of maximum uptake, *M_max_*, shows an increasing trend with temperature and time of aging, reaching near asymptote levels at the highest temperatures and longest time periods of prior thermal aging. The trends are related to the level of curing and cross-link density, which results in lower free volume.
(3) The transition level of uptake, *M_trans_*, indicative of the shift from the faster diffusion-dominated initial regime to the slower second stage, shows an initial increase with the time of prior thermal aging, followed by a decrease.
(4) The ratio *M_trans_*/*M_max_* provides further insight into the relative influence and length of the two primary mechanisms involved—an initial diffusion-dominated regime and a subsequent relaxation-/deterioration-based regime—the latter of which has a far more complex set of competing phenomena, including that of longer-term hydrolysis of the resin, irreversible changes in the network including those of postcuring, and of deterioration through the growth of microcavities and increased fiber–matrix debonding. The trends are in accordance with the phenomena reported in earlier studies related to moisture absorption and temperature, indicating that there are some similarities between sequential exposure and those of simultaneous hydrothermal loading.
(5) The levels of maximum uptake were generally higher in deionized water than in seawater. The opposite trend is noted for uptake levels at the transition point at which the uptake in seawater is about twice that in deionized water, with the difference increasing with the time of prior thermal pre-aging. The highest ratios are seen from 24 h of prior aging onwards, irrespective of the temperature of thermal aging.
(6) Diffusivity, *D*, is directly related to both the temperature and time of prior thermal aging, and the relaxation/deterioration coefficient, *k*, is related through the ratio of mass uptakes, *M_trans_*/*M_max,_* since relaxation is not just a function of moisture uptake-driven mechanisms but rather depends on the evolving network structure of the bulk polymer and the composite and the integrity of the fiber–matrix interface, which is intrinsically dependent on the prior temperature and time of thermal aging. The relaxation/deterioration coefficient, *k*, increases as the ratio *M_trans_/M_max_* decreases, i.e., as the uptake in Stage II becomes larger, i.e., the diffusion-dominated first stage ends earlier in the uptake process. This can be related to the moisture ratio through a single master cure of exponential form.
(7) The investigation shows similarities in the effects of the sequential exposure of thermal aging and subsequent immersion in seawater to that of immersion at elevated temperatures reported more extensively in the literature. It emphasizes the complex interactions between thermal- and moisture-driven phenomena that need to be more comprehensively understood in order to develop accelerated test processes and reliable estimates of long-term response and durability after extreme thermal events. At a first approximation for low levels of prior thermal aging, results from existing elevated temperature immersion can be used as reference levels.
(8) Based on characteristic relationships, the use of moisture uptake levels and especially the ratio *M_trans_*/*M_max_* may provide better metrics for the modeling of long-term performance than that provided by the time of immersion.
(9) The investigation indicates that in general, at temperatures lower than 232 °C, and for the time periods considered, the effects may not be serious enough to warrant removing components from service after exposure as tested, which is of significant practical value in post-extreme event integrity assessment and continued operation.

## Figures and Tables

**Table 1:** Rates of decrease in diffusivity with increase in time to attain *M_trans_*.

Temperature of Thermal Aging (°C)	Rate ×10^−8^ mm^2^/s/Week
66	4.08
93	1.59
121	2.49
149	1.79
177	1.55
204	1.99
232	3.04

**Table 2:** Slopes of diffusivity comparison, m (D_Fickian_ = m D_2-Stage_).

Temperature of Pre-Immersion Thermal Aging (°C)	m	R^2^	Time of Pre-Immersion Thermal Aging (hours)	m	R^2^
66	1.52	0.94	1	1.69	0.97
93	1.51	0.98	2	1.44	0.97
121	1.56	0.99	4	1.52	0.99
149	1.53	0.97	8	1.66	0.96
177	1.50	0.97	16	1.58	0.99
204	1.61	0.98	24	1.72	0.98
232	1.87	0.97	48	1.41	0.96
			72	1.33	0.93

## Data Availability

The data that support the findings of this study are available on request from the corresponding author.
